# Staphylococcus epidermidis Phages Transduce Antimicrobial Resistance Plasmids and Mobilize Chromosomal Islands

**DOI:** 10.1128/mSphere.00223-21

**Published:** 2021-05-12

**Authors:** Lenka Fišarová, Tibor Botka, Xin Du, Ivana Mašlaňová, Pavol Bárdy, Roman Pantůček, Martin Benešík, Pavel Roudnický, Volker Winstel, Jesper Larsen, Ralf Rosenstein, Andreas Peschel, Jiří Doškař

**Affiliations:** a Department of Experimental Biology, Faculty of Science, Masaryk University, Brno, Czech Republic; b Interfaculty Institute of Microbiology and Infection Medicine Tübingen, University of Tübingen, Tübingen, Germany; c Cluster of Excellence EXC2124 Controlling Microbes to Fight Infections, Tübingen, Germany; d Central European Institute of Technology, Masaryk University, Brno, Czech Republic; e Research Group Pathogenesis of Bacterial Infections, TWINCORE, Hannover, Germany; f Institute of Medical Microbiology and Hospital Epidemiology, Hannover Medical School, Hannover, Germany; g Department of Bacteria, Parasites, and Fungi, Statens Serum Institut, Copenhagen, Denmark; Antimicrobial Development Specialists, LLC

**Keywords:** bacteriophages, *Staphylococcus epidermidis*, antibiotic resistance, horizontal gene transfer, pathogenicity islands, transduction

## Abstract

Staphylococcus epidermidis is a leading opportunistic pathogen causing nosocomial infections that is notable for its ability to form a biofilm and for its high rates of antibiotic resistance. It serves as a reservoir of multiple antimicrobial resistance genes that spread among the staphylococcal population by horizontal gene transfer such as transduction. While phage-mediated transduction is well studied in Staphylococcus aureus, S. epidermidis transducing phages have not been described in detail yet. Here, we report the characteristics of four phages, 27, 48, 456, and 459, previously used for S. epidermidis phage typing, and the newly isolated phage E72, from a clinical S. epidermidis strain. The phages, classified in the family *Siphoviridae* and genus *Phietavirus*, exhibited an S. epidermidis-specific host range, and together they infected 49% of the 35 strains tested. A whole-genome comparison revealed evolutionary relatedness to transducing S. aureus phietaviruses. In accordance with this, all the tested phages were capable of transduction with high frequencies up to 10^−4^ among S. epidermidis strains from different clonal complexes. Plasmids with sizes from 4 to 19 kb encoding resistance to streptomycin, tetracycline, and chloramphenicol were transferred. We provide here the first evidence of a phage-inducible chromosomal island transfer in S. epidermidis. Similarly to S. aureus pathogenicity islands, the transfer was accompanied by phage capsid remodeling; however, the interfering protein encoded by the island was distinct. Our findings underline the role of S. epidermidis temperate phages in the evolution of S. epidermidis strains by horizontal gene transfer, which can also be utilized for S. epidermidis genetic studies.

**IMPORTANCE** Multidrug-resistant strains of S. epidermidis emerge in both nosocomial and livestock environments as the most important pathogens among coagulase-negative staphylococcal species. The study of transduction by phages is essential to understanding how virulence and antimicrobial resistance genes spread in originally commensal bacterial populations. In this work, we provide a detailed description of transducing S. epidermidis phages. The high transduction frequencies of antimicrobial resistance plasmids and the first evidence of chromosomal island transfer emphasize the decisive role of S. epidermidis phages in attaining a higher pathogenic potential of host strains. To date, such importance has been attributed only to S. aureus phages, not to those of coagulase-negative staphylococci. This study also proved that the described transducing bacteriophages represent valuable genetic modification tools in S. epidermidis strains where other methods for gene transfer fail.

## Abstract (precis)

Multidrug-resistant strains of S. epidermidis emerge in both nosocomial and livestock environments as the most important pathogens among coagulase-negative staphylococcal species. The study of transduction by phages is essential to understanding how virulence and antimicrobial resistance genes spread in originally commensal bacterial populations.

## INTRODUCTION

Staphylococcus epidermidis is a common commensal inhabitant of the skin and mucosal surfaces in healthy humans and the best characterized coagulase-negative staphylococcal (CoNS) species (1). In recent years, S. epidermidis has gained a lot of attention due to its increasing incidence in nosocomial infections (2). In addition to human diseases, it has also been reported to be the bacterium most frequently isolated from bovine mastitis (3, 4). Resistance to all classes of antibiotics in S. epidermidis has been reported (5), which together with its ability to form a biofilm on indwelling medical devices (6, 7) calls for new drugs or alternative methods for treatment. S. epidermidis is a highly diverse species that, in addition to mutation, also evolves by recombination and readily acquires mobile genetic elements (8). Although S. epidermidis contains few virulence genes in its genome, it acts as a reservoir of antimicrobial resistance genes, including methicillin resistance genes, and is involved in intra- and interspecies horizontal gene transfer associated with the emergence and evolution of drug-resistant strains (5, 9). This is evidenced by the presence of mobile genomic element sequences, including genes associated with pathogenicity islands, which are shared by S. epidermidis and Staphylococcus aureus (10). In S. epidermidis, pathogenicity and resistance islands (SePI and SeRI, respectively) are mostly composite (11) or resemble S. aureus phage-inducible chromosomal islands (PICIs) in their simpler genomic architecture (12–14).

Most of the horizontal gene transfer events in staphylococci are associated with bacteriophages belonging to the family *Siphoviridae* and occur by transduction, which is well studied in S. aureus (15–21). These phages induce lysogenic conversion (22–24), mobilize S. aureus pathogenicity islands (SaPIs) (25–27), and are essential for plasmid transduction (28, 29) and the lateral transfer of chromosomal genes (30, 31), highlighting the importance of siphoviruses for their evolution, which resulted in the highly adaptable pathogenesis strategies of S. aureus. There is a lack of such studies on S. epidermidis. Most knowledge on S. epidermidis siphoviruses is based on their use for phage typing (32, 33), morphological observations (34), lysogeny (35), and comparative genomics studies (36–38).

This work aimed to investigate the role of S. epidermidis phages in the spread of antimicrobial resistance genes. We characterized several new transducing bacteriophages and their capacity to transfer mobile genetic elements. Combined with comparative genomic analyses, these data suggest that S. epidermidis phages have a substantial impact on the fitness of host strains and their evolution.

## RESULTS

### Characterization of S. epidermidis transducing phages.

Phages 27, 48, 456, and 459 were previously used for S. epidermidis phage typing (39, 40) but were not genomically and morphologically characterized, and their role in horizontal gene transfer was not estimated. Here, we compared the above-mentioned phages along with a new phage, E72, which was selected from a set of phages spontaneously induced from 37 clinical S. epidermidis strains based on its optimal growth properties and high-titer production.

**FIG 1:**
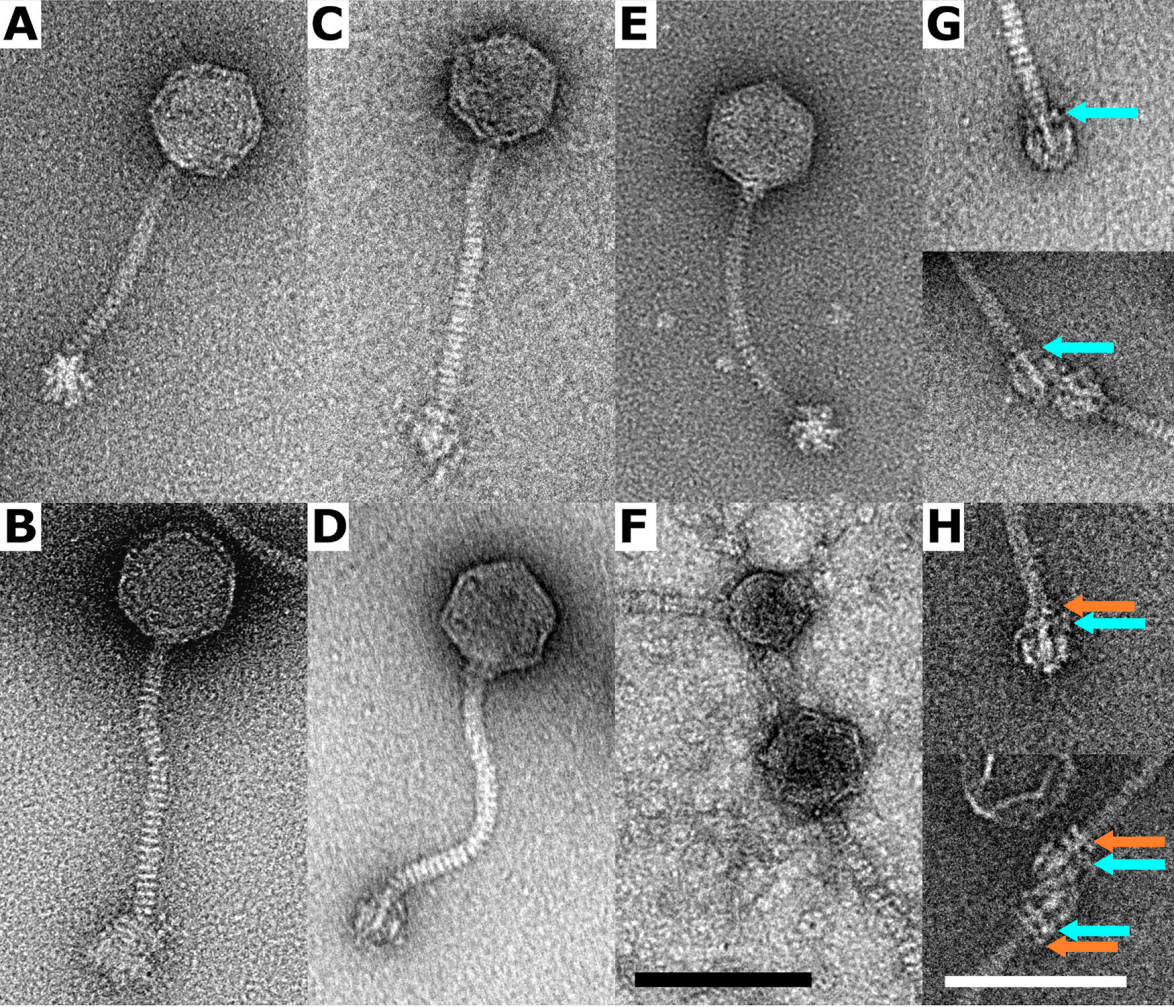
Transmission electron microscope images of negatively stained virions of S. epidermidis phietaviruses. (A) Phage 27; (B) phage 459; (C) phage 456; (D) phage E72; (E) phage 48; and (F) phage 48 and phage 48-derived particle with altered capsid architecture due to interaction with SeCI_SE48_-encoded proteins. (G) Detail of the baseplate of phage E72. In a specific baseplate conformation, a ring of density is present at the end of the tail (cyan arrow). In total, 13 baseplates in this conformation were observed. (H) Detail of the baseplate of phage 456. An extra ring of density is located above the one present in phage E72 (orange arrow). In total, 15 baseplates in this conformation were observed. Bars, 100 nm; black for images A to F (all at the same scale) and white for images G and H.

Transmission electron microscopy showed that all the phages belonged to the family *Siphoviridae* and had similar morphologies (Fig. 1A to E). The diameters of the phage capsids were 65, 61, 68, 70, and 65 (±3) nm in phages 27, 48, 456, 459, and E72, respectively. Their tail lengths were in the range of 145 to 165 nm, and baseplate diameters were in the range of 25 to 30 nm. An exception was the phage 459 baseplate, with a diameter of 37 nm and a different, fuzzier shape than the rest of the studied phages (Fig. 1B).

**FIG 2:**
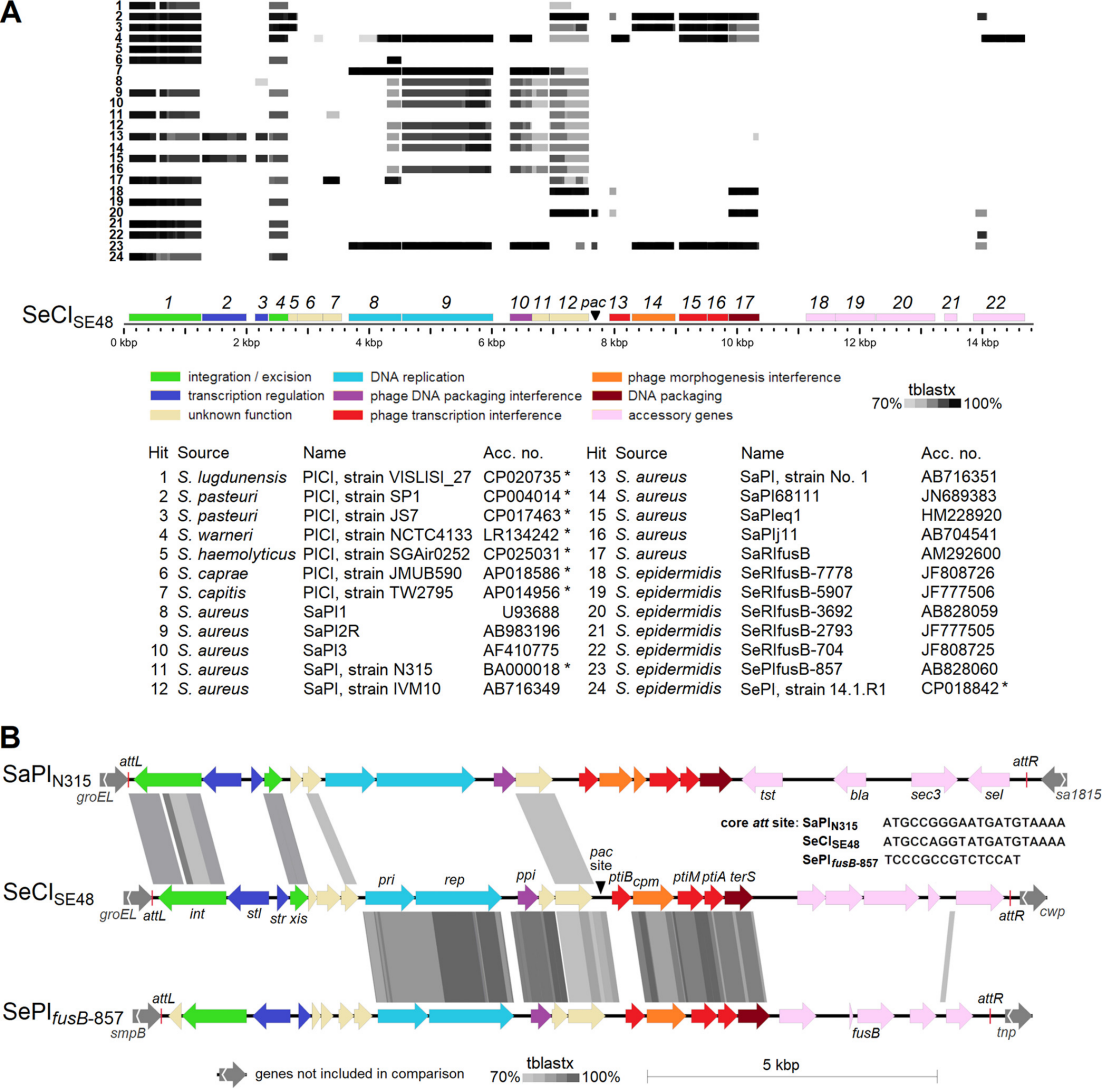
Comparison of phage-inducible chromosomal island SeCI_SE48_ with selected chromosomal islands. (A) Islands identified in staphylococcal genomes available in GenBank. SeCI_SE48_ genes are color coded according to their product function, and the corresponding gene product number is given above them. Chromosomal islands obtained from complete genomes of staphylococcal strains as 20-kb sequences starting with the integrase gene are indicated with asterisks. (B) Detailed comparison of SeCI_SE48_ with SePI*_fusB_*_-857_ and SaPI_N315_. Genes are color coded according to their product function. Identity based on tblastx comparison is represented by shaded boxes with a cutoff of 70%.

In phage 48 propagated on strain SE48, small-headed phage-like particles with a capsid diameter of 48 nm were observed (Fig. 1F). Liquid chromatography-tandem mass spectrometry (LC-MS/MS) analysis of phage 48 virions identified 12 phage-encoded proteins (Table S1). In addition, a capsid morphogenesis protein encoded by the *cpm* gene (*gp14*; GenBank accession no. QRX38697) of the newly discovered chromosomal island of S. epidermidis strain SE48 was detected. This points to its effect on capsid remodeling associated with island mobilization, which was first observed and described in S. epidermidis here. The chromosomal island, designated SeCI_SE48_, exhibited similarities in architecture and the integrase gene with S. epidermidis fusidic acid resistance islands (14) (Fig. 2).

10.1128/mSphere.00223-21.1 TABLE S1 Mass spectrometry identification of proteins forming particles of phage 48 propagated on S. epidermidis strain SE48. Structural proteins of phage and phage-derived small-headed particles purified in CsCl gradient were determined by LC-MS/MS analysis. Download Table S1, PDF file, 0.05 MB. Copyright © 2021 Fišarová et al. 2021 Fišarová et al. https://creativecommons.org/licenses/by/4.0/ This content is distributed under the terms of the Creative Commons Attribution 4.0 International license.

**FIG 3:**
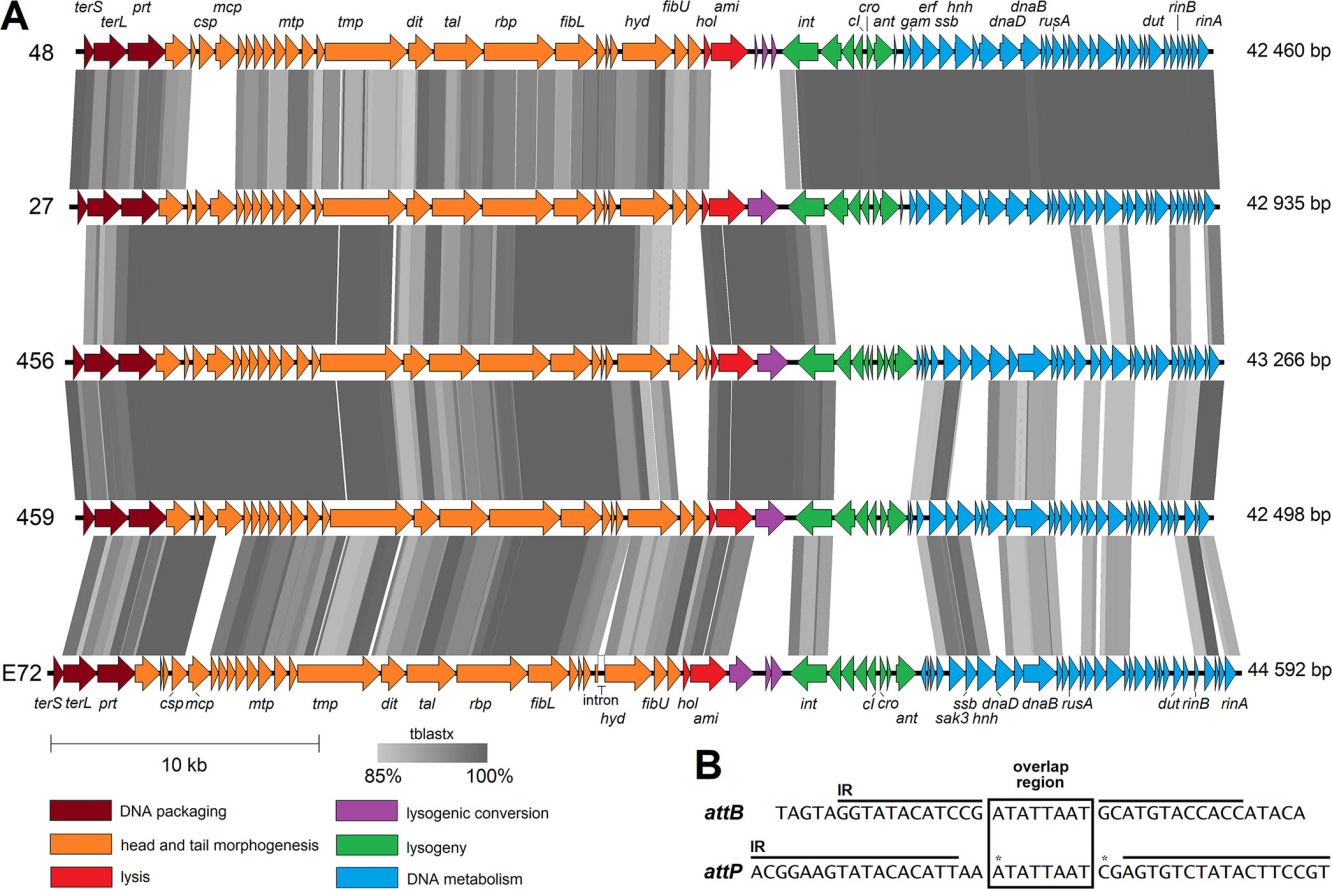
Whole-genome sequence-based comparison of S. epidermidis phages 48, 27, 456, 459, and E72. (A) Comparison of the phage genomes. Predicted genes are indicated with full arrows colored according to their modular affiliation. Shaded boxes show the amino acid sequence identity levels between genomes based on tblastx comparison. The genes encoding the following putative products are depicted: terminase small subunit (*terS*), terminase large subunit (*terL*), portal protein (*prt*), capsid scaffolding protein (*csp*), major capsid protein (*mcp*), major tail protein (*mtp*), tape measure protein (*tmp*), distal tail protein (*dit*), tail-associated lysin (*tal*), receptor-binding protein (*rbp*), lower tail fiber (*fibL*), cell wall hydrolase (*hyd*), upper tail fiber (*fibU*), holin (*hol*), amidase (*ami*), integrase (*int*), CI-like repressor (*cI*), Cro-like repressor (*cro*), antirepressor (*ant*), Gam-like nuclease inhibitor (*gam*), ERF-like single-strand annealing protein (*erf*), Sak3-like single-strand annealing protein (*sak3*), single-stranded DNA binding protein (*ssb*), HNHc nuclease (*hnh*), replication protein DnaD (*dnaD*), replicative helicase DnaB (*dnaB*), resolvase (*rusA*), dUTP diphosphatase (*dut*), transcription activator RinB (*rinB*), transcription regulator RinA (*rinA*). (B) Consensus sequences of *attB* sites of S. epidermidis strains and *attP* sites of S. epidermidis phietaviruses. The overlap region is indicated by the box. Imperfect inverted repeats are indicated with black lines above the sequences. The positions of the substitutions (G and A, respectively) in phage 48 are indicated with asterisks. The hybrid *attL* attachment site (TAGTAGGTATACATCCGATATTAATAGAGTGTCTATACTTCCGT) of strain SE48 lysogenized with phage 48, determined by PCR amplicon *mer*-*int* sequencing (Table 4), corresponds perfectly to the respective portions of *attB* of strain SE48 and *attP* of phage 48.

**FIG 4:**
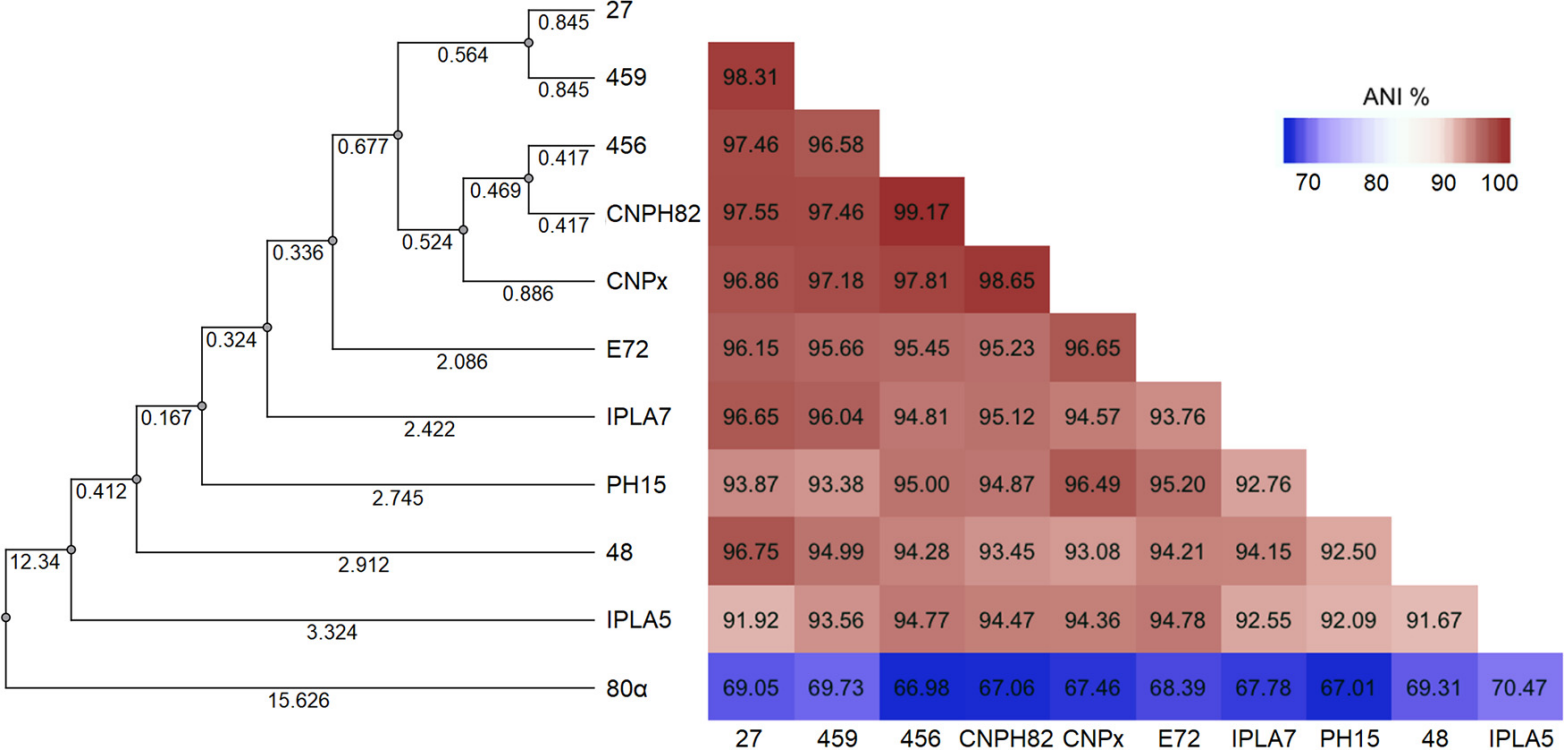
Dendrogram of interphage relatedness of S. epidermidis phietaviruses and S. aureus phietavirus 80α. The interphage relatedness was calculated with the OrthoANI algorithm, and clustered using unweighted pair group method using average linkages (UPGMA). The corresponding pairwise nucleotide identity heat map is shown, with percentage values.

The genomes of all the phages studied were assembled using short Illumina reads. They were arranged in functional modules, and their size was slightly above 40 kb (Fig. 3A). As no defined termini of genomic DNA were obvious from the Illumina read alignment, phages most likely use a headful packaging mechanism and have circularly permuted genomes with redundant ends (41). The analyzed S. epidermidis phages exhibited high average nucleotide identity (94 to 98%) to each other and 67 to 70% identity to S. aureus phietavirus 80α, suggesting that *Phietavirus* is a common phage genus in both staphylococcal species (Fig. 4). Comparison of the genomes of phages 27, 456, and 459 revealed high similarity in the virion structure module. Moreover, phages 48 and 27 shared almost identical lysogeny and DNA metabolism modules, while the other modules differed (Fig. 3A; also, see Table S2 in the supplemental material).

10.1128/mSphere.00223-21.2 TABLE S2 Annotation of predicted S. epidermidis phage 48-encoded proteins compared to phages 27, 456, 459 and E72. Download Table S2, PDF file, 0.1 MB. Copyright © 2021 Fišarová et al. 2021 Fišarová et al. https://creativecommons.org/licenses/by/4.0/ This content is distributed under the terms of the Creative Commons Attribution 4.0 International license.

**FIG 5:**
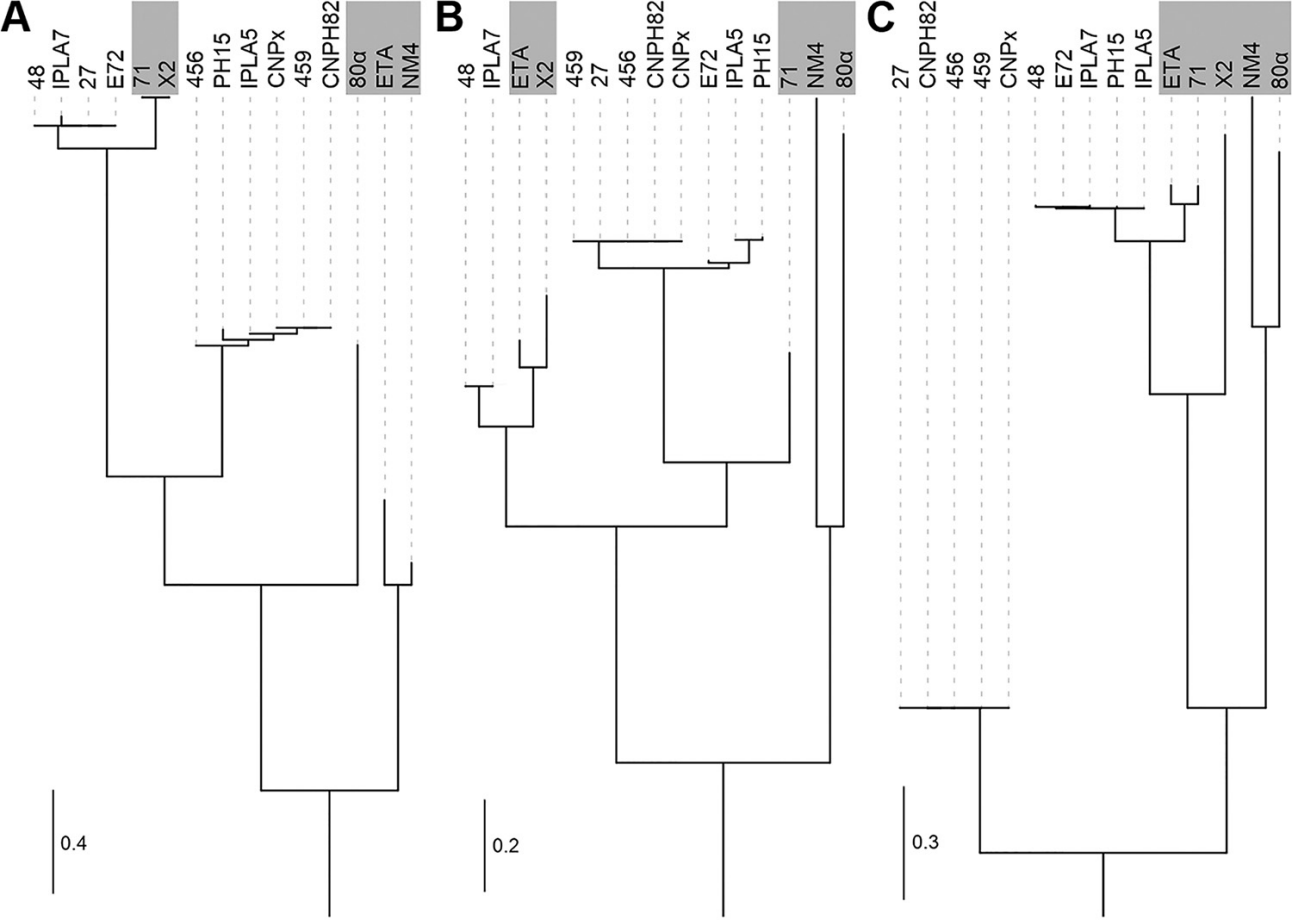
Phylogenetic trees based on multiple-sequence alignment of three phage proteins illustrating different phylogenetic clades within S. aureus and S. epidermidis phietaviruses and the modular character of their genomes. (A) Terminase small subunit (TerS); (B) capsid scaffolding protein (Csp); (C) major capsid protein (Mcp). S. aureus phages ETA, 71, X2, 80α, and NM4 are outlined with a gray background. The trees are the results of the maximum-likelihood-based inference of phylogenetic trees with Smart Model Selection.

When particular functional genome modules in their respective order were focused on, the following differences were observed. A comparison of the amino acid sequences of the terminase small subunit (TerS), which tends to be one of the least conserved phage proteins (42), divides known S. epidermidis phietaviruses into two subgroups, where phages 27, 48, and E72 represent one and phages 456 and 459 represent the other (Fig. 5A). The genes encoding the capsid scaffolding protein (Csp) and the major capsid protein (Mcp) exhibited notable differences when phage 48 is compared with other phages analyzed in this study. Based on the Csp sequence analysis, all of the studied phages except phage 48 grouped into one cluster (Fig. 5B), while they formed two distinct clusters based on Mcp analysis (Fig. 5C). Phylogenetic trees based on alignments of TerS, Csp, and Mcp show reticulate relationships and mosaicism among phietaviruses and the relatedness of S. epidermidis phages to those of S. aureus, especially 71 and X2 (43) and ETA (44), while S. aureus phages NM4 (45) and 80α (46) are more distantly related (Fig. 5).

Baseplate structural components important for host recognition, i.e., receptor-binding proteins (RBP) encoded by the *rbp* gene of phages 459 and 27, are the same and almost identical to the RBP of phages E72 (99.4% identity) and 456 (99.3% identity). They exhibit 97.7% identity to the RBP of phage 48. Similarly, the lower tail fiber protein (FibL) is conserved, exhibiting ≥98% identity. The RBP of S. aureus phietavirus 80α (21) exhibits about 25% identity and 37% similarity to those of studied S. epidermidis phages, and FibL exhibits about 28% identity and 40% similarity. Another protein associated with host range, the cell wall hydrolase (Hyd) encoded by the *hyd* gene, hypothesized to be associated with the baseplate (21), varies slightly across all S. epidermidis phages (Table S2). Besides this, a noncoding intron was predicted in the *hyd* gene of phage E72 (Fig. 3A). Genes for upper tail fiber (*fibU*) and a hypothetical protein following the *hyd* gene are replaced by three diverse genes in phage 456. This is manifested as an extra ring of density above the baseplate (Fig. 1G and H).

The lysis module comprising genes for holin (*hol*) and amidase (*ami*) in phage 48 differs significantly from the other S. epidermidis phages (Fig. 3A; Table S2). Its amidase gene has only about 50% identity to other S. epidermidis phages but exhibits 78% identity to S. aureus phage B236 (23).

In the S. epidermidis phages studied here, no known genes associated with lysogenic conversion were identified in the region following the lysis genes (Fig. 3A). Phages 27, 456, and 459 harbor the same putative endonuclease gene in this region. In E72, a gene encoding a different putative endonuclease (*gp30*) is located here and is followed by a gene encoding a hypothetical protein and a gene for a putative membrane-associated protein (*gp32*). In this region, the phage 48 genome contains three genes, which encode short hypothetical proteins (Table S2).

The S. epidermidis phages studied here have almost identical integrases of the serine-type family (Table S2). An integrase gene with 95% identity was found in a prophage sequence of S. epidermidis NCTC 13924 (GenBank accession no. NZ_LR134536). In the laboratory-lysogenized strains prepared in this study, the genomes of all the phages integrated into the same site in the gene encoding a FAD-dependent oxidoreductase (GenBank accession no. WP_080035152 in strain 1457), which in S. aureus was annotated as a probable pyridine nucleotide-disulfide oxidoreductase family protein, resulting in the split of this gene into two open reading frames (ORFs). The sequence ATATTAAT of the assumed overlap region, where the crossover between *attB* and *attP* occurs, is flanked by imperfect inverted repeats (Fig. 3B).

### Phage growth characteristics.

The host range determined on 35 S. epidermidis and two S. aureus strains (Table 1) and 35 field isolates of seven CoNS species showed that the phages studied here and the previously sequenced phage PH15 (36) were species specific for S. epidermidis alone. Most of the susceptible strains were isolated in the 1970s (39, 47), while recent S. epidermidis isolates were predominantly resistant (17, 48–52) (Table 1). The broadest host range on S. epidermidis strains (*n* = 35) was that of phage E72 (34%), followed by 456 (31%) and 27 (26%), and the narrowest were the host ranges of phages 48 and 459 (both 17%). Phage susceptibility testing was conducted by the plate method; however, the ability of phages to effectively propagate on hosts may also depend on the type of culture, i.e., liquid or solid. Strain 1457 did not exhibit sensitivity to phages 27, 48, and 456 (Table 1), but when these phages were incubated with strain 1457 in liquid medium (meat-peptone broth [MPB] or tryptone soy broth [TSB]), they were able to propagate, which enabled their use in transduction experiments. No correlation was found between lytic ability and adsorption kinetics. After 15 min, a portion of the studied S. epidermidis phages remained unadsorbed for both phage-resistant and -susceptible strains.

**TABLE 1:** Host range of S. epidermidis phages 27, 48, 456, 459, and E72 characterized in this study and phage PH15 described previously

Bacterial strain^*a*^	Reference or source^*b*^	Lysis results for phage^*c*^:					
		PH15	27	48	456	459	E72
S. epidermidis SE15	39	C, C, C	C, C, C	S, C, C	C, C, C	−, −, −	S, C, C
S. epidermidis SE27	39	C, C, C	C, C, C	S, C, C	S, C, C	−, −, −	C, C, C
S. epidermidis A6C	39	C, C, C	C, C, C	S, C, C	C, C, C	−, P, S	C, C, C
S. epidermidis A9C	39	C, C, C	C, C, C	S, C, C	S, C, C	P, S, C	C, C, C
S. epidermidis SE37	39	−, −, −	−, −, −	−, −, −	−, −, −	−, −, −	−, −, −
S. epidermidis SE155	39	−, −, −	−, −, −	−, −, −	−, −, −	−, −, −	−, −, −
S. epidermidis SE48	47	−, −, −	P, P, S	C, C, C	−, −, −	−, −, −	−, −, L
S. epidermidis SE456	47	−, −, −	−, −, −	−, −, −	C, C, C	−, −, −	P, P, S
S. epidermidis SE459	47	P, P, S	P, S, C	P, S, C	P, S, C	S, C, C	P, S, C
S. epidermidis SE471	47	−, −, −	−, −, −	−, −, −	C, C, C	−, −, −	C, C, C
S. epidermidis 1457	49	−, −, −	−, −, −	−, −, −	−, −, −	P, S, C	S, C, C
S. epidermidis 1457 (SeCI_SE48_)	This work	−, −, −	−, −, −	−, −, −	−, −, −	−, −, −	−, −, −
S. epidermidis RP62A	50	−, −, −	−, −, −	−, −, −	−, −, −	−, −, −	−, −, −
S. epidermidis O−47	52	−, −, −	−, −, −	−, −, −	−, −, −	−, −, −	−, −, −
S. epidermidis Tü 3298	48	−, −, −	−, L, L	−, −, −	−, −, −	−, −, −	−, −, L
S. epidermidis CCM 50	CCM	−, −, −	P, S, C	−, −, −	P, S, C	−, −, P	−, −, −
S. epidermidis CCM 4187	CCM	−, −, −	P, P, S	−, −, −	P, P, S	−, −, −	−, −, L
S. epidermidis CCM 2343	CCM	−, −, −	P, S, C	−, −, −	P, P, S	−, P, P	−, −, −
S. epidermidis CCM 4418	CCM	−, −, −	−, −, −	−, −, −	C, C, C	−, −, −	−, L, L
S. epidermidis CCM 7844	CCM	−, −, −	−, −, −	−, −, −	−, −, −	−, −, −	−, S, C
S. epidermidis CCM 7221	CCM	−, −, −	−, −, −	−, −, −	−, −, −	−, −, −	−, −, −
S. epidermidis CCM 2124	CCM	−, −, L	−, −, L	−, −, −	−, −, −	−, −, −	−, −, P
S. epidermidis CCM 4505	CCM	−, −, −	−, −, −	−, −, −	−, −, −	−, −, −	−, P, S
S. epidermidis 4	UPOL	−, −, −	−, −, L	−, −, −	−, −, −	−, −, −	S, C, C
S. epidermidis 2	UPOL	−, −, −		−, −, −	−, −, −	−, −, −	−, −, L
S. epidermidis 18	UPOL	−, −, −	−, −, L	−, −, −	−, −, −	−, −, −	−, −, L
S. epidermidis 58	UPOL	−, −, −	−, −, −	−, −, −	−, −, −	−, −, −	−, −, −
S. epidermidis 221−1	UPOL	−, −, −	−, −, L	−, −, −	−, −, −	−, L, L	L, L, L
S. epidermidis 257−2	UPOL	−, −, −	−, −, −	−, −, −	−, −, −	−, −, −	−, −, L
S. epidermidis 341−5	UPOL	−, −, −	−, −, −	−, −, −	−, −, −	−, −, −	−, −, −
S. epidermidis 348−6	UPOL	−, −, −	−, −, −	−, −, −	−, −, −	−, −, −	−, −, −
S. epidermidis 352−7	UPOL	−, −, −	−, −, −	−, −, −	−, −, −	−, −, −	−, −, −
S. epidermidis 354−8	UPOL	−, −, −	−, −, −	−, −, −	−, −, −	−, −, −	−, −, −
S. epidermidis 357−9	UPOL	−, −, −	−, −, −	−, −, −	−, −, −	−, −, −	−, −, L
S. epidermidis 465−4	UPOL	−, −, −	−, −, −	−, −, −	−, −, −	−, −, −	−, −, L
S. aureus RN4220	51	−, −, −	−, −, −	−, −, −	−, −, −	−, −, −	−, −, −
S. aureus 8325−4	17	−, −, −	−, −, −	−, −, −	−, −, −	−, −, −	−, −, −

a “SE” in a strain name indicates a propagating strain for phages.

b CCM, Czech Collection of Microorganisms, Masaryk University (Brno, Czech Republic); UPOL, Department of Microbiology, Faculty of Medicine and Dentistry, Palacky University (Olomouc, (Czech Republic) (methicillin-resistant S. epidermidis human clinical isolates).

c The result of each of the three replicates is given. C, confluent lysis; S, lysis; P, individual plaques; L, lysis from without (early bacterial lysis induced by high-multiplicity virion adsorption without phage propagation or abortive infection, manifested by a turbid zone); −, no lysis. For the C, S, P pattern, the formation of single plaques at higher phage dilution was confirmed for all sensitive strains.

### Mobilization and transfer of a chromosomal island.

**FIG 6:**
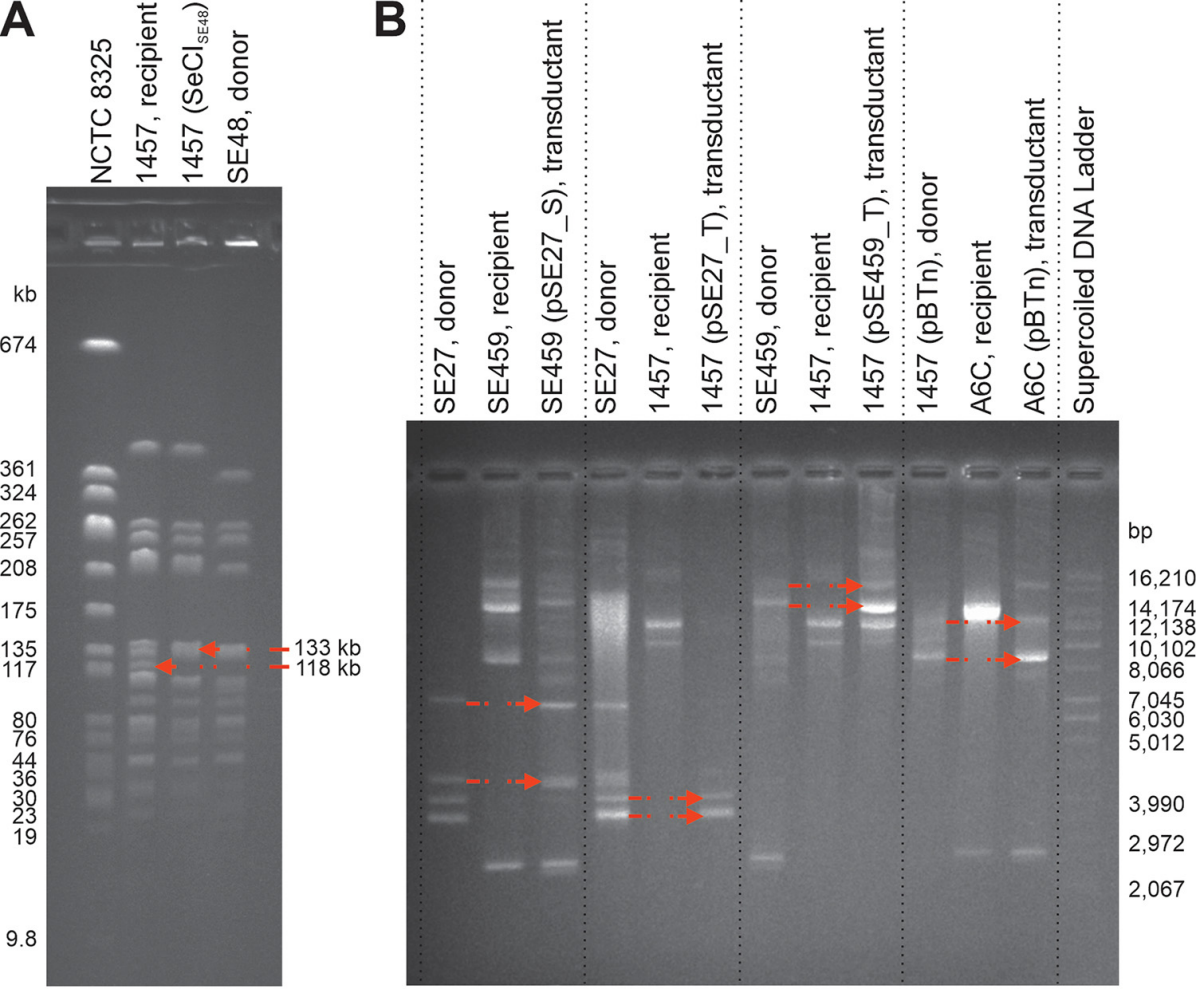
Analysis of transduced mobile elements by agarose gel electrophoresis. (A) PFGE separation of SmaI macrorestriction fragments of genomic DNA of recipient S. epidermidis strain 1457 before and after the insertion of SeCI_SE48_, which was transduced by phage 48 from donor S. epidermidis strain SE48. The red arrows show a 118-kb fragment of the recipient and the corresponding fragment in the transductant enlarged to 133 kb due to SeCI_SE48_ integration. S. aureus strain NCTC 8325 was used as a marker. (B) Plasmid profile of the S. epidermidis donor, recipient, and transductant strains in the transductions of plasmids pSE27_S, pSE27_T, pSE459_T, and pBTn, respectively. Detailed information is given in Table 2. The transferred plasmids in their supercoiled and relaxed forms are indicated with arrows.

While the sequencing data of phage 48 propagated on S. epidermidis strain SE48 were being analyzed, the sequence of a new chromosomal island SeCI_SE48_ was identified. The distribution of its coverage by short Illumina sequencing reads showed that the island was packaged in the phage virions in linear form from the *pac* site by a headful mechanism. The *pac* site of SeCI_SE48_ was predicted at the position between *gp12*, which encodes an unknown protein, and *ptiB*, which encodes a phage transcription inhibitor (Fig. 2B). Using the sequencing data of phage 48, the ratio of mean coverage of SeCI_SE48_ and phage 48 DNA sequences was 1.5 × 10^−2^, which indicates that SeCI_SE48_ is packaged at high frequency. In the host chromosome, SeCI_SE48_ is integrated into *att*B at the 3′ end of the *groEL* gene (Fig. 2B). As no antimicrobial resistance factors encoded by SeCI_SE48_ were predicted, the transduction of this island by phage 48 into the recipient strain 1457 was performed without antibiotic selection (Table 2), and the transductants were selected by pulsed-field gel electrophoresis (PFGE) analysis (Fig. 6A). Strain 1457 does not contain any chromosomal island adjacent to the *groEL* gene. After the transfer to strain 1457, the SeCI_SE48_ integrated at the 3′ end of *groEL* gene, which was not altered due to its integration, as the last 18 bp of the gene overlap the SeCI_SE48_ *att* site. PICIs with the same type of integrase and excisionase are localized downstream of the *groEL* gene in several staphylococcal species, including S. aureus, Staphylococcus lugdunensis, Staphylococcus pasteuri, Staphylococcus warneri, and Staphylococcus haemolyticus (Fig. 2A). Of the chromosomal islands described above, SeCI_SE48_ exhibited the highest similarity with SePI_fusB-857_, which encodes fusidic acid resistance (12), and a different integrase (Fig. 2B).

**TABLE 2:** Transductions of S. epidermidis mobile genetic elements

Donor	Mobile genetic element			Transduction system			
	Name	Size (bp)	Resistance gene(s)	Recipient	Phage	Selection antibiotic	Frequency
SE27	pSE27_S	4,397	*aad(6)*	SE459	27	Streptomycin	NA^*a*^
SE27	pSE27_T	4,439	*tetK*	1457	48	Tetracycline	5.0 × 10^−4^
SE48	SeCI_SE48_	14,835		1457	48		NA^*b*^
SE459	pSE459_T	18,761	*tetK*	1457	459	Tetracycline	1.6 × 10^−7^
1457 (pBTn)	pBTn	11,256	*bla, erm, cat*	A6C	48	Chloramphenicol	5.4 × 10^−5^
					456	Chloramphenicol	3.7 × 10^−5^
					459	Chloramphenicol	6.9 × 10^−5^
					E72	Chloramphenicol	6.1 × 10^−5^
1457 (pBTn)	pBTn	11,256	*bla, erm, cat*	1457	48	Chloramphenicol	6.1 × 10^−6^
					456	Chloramphenicol	3.9 × 10^−7^
					459	Chloramphenicol	2.0 × 10^−7^
					E72	Chloramphenicol	9.3 × 10^−7^

a NA, not applicable due to emergence of spontaneous streptomycin-resistant mutants.

b NA, not applicable due to no antibiotic selection.

The burst size of phage 48 on the transductant strain 1457(SeCI_SE48_) was significantly decreased (4.3-fold) compared to that on wild-type strain 1457. SeCI_SE48_ integration also led to a loss of sensitivity of strain 1457(SeCI_SE48_) to phages 459 and E72 (Table 1).

### Plasmid transductions.

All bacterial sequences were obtained using a hybrid assembly of Illumina reads and long Oxford Nanopore reads. All the propagating strains differing in their sequence types (STs) used here carry one or more plasmids, some of which determine antibiotic resistance (Table 3). Phages 27, 48, and 459 transferred naturally occurring plasmids (Table 2; Fig. 6B). Then, a chloramphenicol resistance-encoding plasmid, pBTn (53), electroporated into S. epidermidis strain 1457 was transferred by all the phages except phage 27 into S. epidermidis strains 1457 and A6C (Table 2; Fig. 6B). The transduction efficiency differed according to the phage and recipient strain used (Table 2). Interspecies plasmid transfer from strains SE27, SE48, SE456, and SE459 by phages able to propagate on them (listed in Table 1) into other staphylococcal species, including three strains of Staphylococcus sciuri, two strains of Staphylococcus chromogenes, two strains of Staphylococcus xylosus, one strain each of Staphylococcus petrasii and Staphylococcus simulans, and seven S. aureus strains, was not successful (data not shown).

**TABLE 3:** Basic genomic properties of S. epidermidis propagation strains and their plasmids

Strain	MLST (CC)^*a*^	Plasmid name	GenBank accession no.	Size (bp)	AR gene(s)^*b*^	Most similar GenBank entry(ies) (organism), % identity^*c*^
SE27	ST190 (CC5)	pSE27_T	MW364977	4,439	*tetK*	CP033733 (S. hominis), 100; CP038249 (*S. warneri*), 100; GQ900445 (S. aureus), 99; AE015930 (S. epidermidis), 99
		pSE27_S	MW364976	4,397	*aad(6)*	MH090916 (S. epidermidis), 100; CP014406 (S. aureus), 100; AP009489 (Macrococcus caseolyticus), 100
SE48	ST215^*d*^ (CC14)	pSE48_1	NZ_CP066304	8,255	*fmhA*, *abc*	Novel
		pSE48_2	NZ_CP066305	1,289	*ant(4)*	GQ900442 (S. aureus), 99
SE456	ST73 (CC73)	pSE456_1	MW364978	43,075	*abc*	Novel
		pSE456_2	MW364979	13,277	*abc*	Novel
		pSE456_3	MW364980	8,330	*abc*	Novel
		pSE456_4	MW364981	2,348		CP047868 (S. aureus), 100
SE459	ST14 (CC14)	pSE459_1	MW364982	20,983		Novel
		pSE459_T	MW364985	18,761	*tetK, abc*	Novel
		pSE459_2	MW364983	11,465		Novel
		pSE459_3	MW364984	2,789		Novel
1457	ST86 (CC14)	p1457	NZ_CP020462	15,142		NA

a MLST, multilocus sequence type.

b Known or putative antibiotic resistance gene. AR, antibiotic resistance. *tetK*, gene encoding tetracycline efflux MFS transporter; *aad(6)*, gene encoding streptomycin aminoglycoside 6-adenyltransferase; *fmhA*, gene encoding aminoacyltransferase possibly involved in methicillin and lysostaphin resistance; *ant(4)*, gene encoding aminoglycoside nucleotidyltransferase; *abc*, gene encoding ABC transporter (may be involved in drug resistance; multiple *abc* genes are present in appropriate plasmids).

c If more than one bacterial species is present, one plasmid is given for each of them; plasmids with overall nucleotide sequence identity less than 75% are noted as novel. NA, not performed.

d Nearest ST; no match with the *arcC* gene was found.

### Application of transducing phages for genetic studies.

Our results showed that the transfer of pBTn via electroporation to the 37 clinical S. epidermidis strains from University Hospital Tübingen tested here failed in 26 cases (70.3%) (Table S3). Therefore, effectively transducing S. epidermidis phages could be considered another laboratory tool for the genetic manipulation of S. epidermidis. Phage E72 exhibited high transducing efficiency (up to 10^−4^) by transferring pBTn from strain 1457 (pBTn) to 21 of 37 (56.8%) S. epidermidis strains, including 10 of 19 (52.6%) strains that could not receive pBTn using electroporation or transduction by phage 187 (18) (Table S3). Moreover, we observed that phage E72 could be propagated to a high titer of ∼10^11^ PFU/ml, which is an advantage for successful genetic manipulation using transduction (54).

10.1128/mSphere.00223-21.3 TABLE S3 Transfer of plasmid pBTn via electroporation and transduction mediated by phages 187 and E72. The ratio of transduction is presented as the number of transductants per milliliter of phage lysate relative to the transducing phage titer; each transduction was carried out in triplicate, and the average rate is provided. Download Table S3, PDF file, 0.3 MB. Copyright © 2021 Fišarová et al. 2021 Fišarová et al. https://creativecommons.org/licenses/by/4.0/ This content is distributed under the terms of the Creative Commons Attribution 4.0 International license.

## DISCUSSION

A detailed genomic and phenotypic analysis of five S. epidermidis phietaviruses allowed us to assess their transduction potential and putative impact on the evolution of S. epidermidis. Published phietavirus genomes are exclusive to S. aureus and S. epidermidis phages (searched using NCBI Virus). Before this study, phietaviruses amounted to nine of the 13 S. epidermidis siphoviral genomes available in NCBI Virus, which together with the phages described here makes this genus an abundant group of temperate phages in S. epidermidis.

Our results confirmed that generalized transduction in S. epidermidis is mediated by temperate phages using the headful mechanism for DNA packaging (55). Genomes of the studied phages had no obvious *pac* site, thus resembling S. aureus phage 11 (56). During the packaging initiation, the DNA is probably cut not at a precise location but at scattered locations within a large region of up to several kilobases (57). This relaxed specificity enables the packaging of heterogeneous DNA with pseudo-*pac* sites into the virion capsids. Differences in pseudo-*pac* site homology may therefore lead to an altered frequency of transduction. A phage-encoded small terminase subunit (TerS) is essential for phage genome and plasmid packaging into the capsid, but it is not required for the packaging of phage-inducible islands that encode distinct TerS recognizing their own specific *pac* site (58). We hypothesize that the high efficiency of plasmid transduction by phages 27, 48, and E72 is determined by the same TerS type that is distinct from the other studied phages.

The phage susceptibility of the tested bacterial strains does not always correlate with the adsorption rate of the phage. Therefore, it may depend on many postadsorption factors, as was recently reviewed for S. aureus (59). For generalized transduction, the permissivity of a recipient strain to productive phage infection is not required for receiving foreign DNA; however, cell wall penetration is a necessary prerequisite (29). Differences in baseplate structural proteins may relate to the different ability to infect the host (60). Phage 456 differed from other S. epidermidis phages except for phage CNPx (61) in several baseplate component-encoding genes, including *fibU* for upper tail fiber protein, which is not essential for either infectivity or assembly of the baseplate in phage 80α (21). We assume it may play a supporting role in host recognition. Phages 456 and E72, which have similar RBP, exhibited the broadest but not identical host ranges. In addition, phage E72 was able to lyse some of the strains resistant to all other tested phages. This could be connected with a small transmembrane domain protein, encoded by *gp24* localized in the tail structure region, which could play a role in the host cell envelope penetration via holin regulation.

Horizontal gene transfer in S. aureus is common within clonal lineages, while transfer between them is rare due to specific defense mechanisms, such as restriction-modification systems (62). However, we were able to show that phages 27 and 48 transferred plasmids from S. epidermidis CC5 to CC14. Based on our previous findings on transduction to S. aureus strains insensitive to the lytic action of a transducing phage (29), we analyzed interspecies transfer; however, we found no evidence for such an ability of any of the phages. Nevertheless, the plasmids that were transferred between S. epidermidis clonal complexes show high overall identity to plasmids of Staphylococcus hominis, *S. warneri*, S. aureus and Macrococcus caseolyticus (Table 3). Only a few previous studies demonstrated plasmid transfer between different staphylococcal species using electroporation (63) or by transduction (64).

To compare the transduction ability of the tested phages, the transfer frequency of the 11.2-kb chloramphenicol resistance plasmid pBTn was assessed. The transduction rates of the tested phages differed on strain 1457 but not on strain A6C, where all the transducing phages transferred pBTn with a higher rate. Only phage 27 was incapable of transduction into strains 1457 and A6C, which may be connected with host surface structures, as was demonstrated in streptococci (65). In naturally occurring plasmids, more efficient transduction of smaller ones was observed, as was described before (66).

The transfer of PICIs has already been well described for S. aureus (SaPIs), but this study provides the first evidence of such a transfer in S. epidermidis. Recently, PICIs have also been predicted in genomes of other Gram-positive cocci (67). They are only distantly related to each other and to S. aureus SaPIs, but they share a genome organization and content. Therefore, they represent convergent evolution that suggests their high selective value. In the previously described staphylococcal PICIs, the accessory genes encode toxins and/or antibiotic resistance determinants (12, 13, 68), but SeCI_SE48_ harbors genes for putative membrane proteins. SeCI_SE48_ is integrated at the 3′ end of the host *groEL* gene, similar to S. aureus and S. epidermidis PICIs with the same type of integrase (12–14).

The role of the PICI-encoded proteins PtiA, PtiM, and PtiB in the inhibition of phage late transcription through the interaction with phage transcriptional regulator RinA was demonstrated previously (69). The same mechanism was possibly responsible for the decreased phage susceptibility in strain 1457 after SeCI_SE48_ was integrated into its chromosome. An alteration of the phage life cycle by PICI, leading to the formation of small-headed virions that are unable to carry the entire phage genome, was described (25). In staphylococcal phage 80α, the capsid size change is caused by SaPI1 proteins redirecting the phage capsid architecture from T=7 to T=3 (70). The observed formation of small-headed virions in phage 48 suggests a similar architecture shift in S. epidermidis. The primary sequence and predicted secondary structure of the SeCI_SE48_-encoded protein, involved in the capsid assembly of small-headed virions, differs from those in SaPI1 (70). This suggests that SeCI_SE48_ uses a different mechanism of phage capsid resizing.

The homologues of phage 80α-encoded PICI derepressors, such as dUTPase, Sri, and ORF15, are encoded by genes in the DNA metabolism module and play an essential role in chromosomal island mobilization (27, 71). In S. epidermidis phages 27 and 48, homologues of these PICI mobilization determinants are identical (Fig. 3), but these phages differ significantly in their capsid proteins. While the minor capsid protein has been shown to not affect plasmid or PICI transduction (72), the role of other capsid proteins has been only partially described (70). In phage 48, the capsid scaffolding protein was distinct from all the tested phages. High-frequency transducing phages 48 and E72 encode almost identical major capsid proteins that are very different from all other phages. Thus, the capsid proteins seem to play an important role not only in the mobilization of islands but also in the transfer of plasmids, where the successful transduction is dependent on a complex of genetic determinants of both the mobile element and the phage.

The transfer of plasmids to bacterial cells is an important technique used in research in S. epidermidis. However, far fewer clinical S. epidermidis than S. aureus isolates can be transformed by electroporation, and the efficiency is orders of magnitude lower, despite attempts at optimization (73, 74). While there are many studies of phage-mediated plasmid transduction in S. aureus, there is a lack of similar studies in S. epidermidis. Recently, Winstel et al. (18) established a plasmid transfer method using phage 187, which is capable of efficient transduction to CoNS strains and specific S. aureus strains with CoNS-type wall teichoic acid. The phages used in our study also exhibited a high efficiency of plasmid transduction in strains where phage 187 failed (Table S3). They even transferred relatively large plasmids that generally cannot be easily transferred via electroporation or by phage 187. Therefore, the phages described in this study, especially E72 and 48, could be considered suitable laboratory tools for the transduction of plasmids and chromosomal islands to S. epidermidis strains, which could promote future CoNS research.

## MATERIALS AND METHODS

### Phages and bacterial strains.

Bacteriophages PH15 and 27 (39), phages 48, 456, and 459 (40, 47), and their propagating S. epidermidis strains SE15, SE27, SE48, SE456, and SE459 were obtained from V. Hájek (Palacký University, Olomouc, Czech Republic). Thirty-seven clinical S. epidermidis isolates were obtained from the Medical Microbiology department of the University of Tübingen (Table S3). Phage E72 was induced from S. epidermidis strain E72, a clinical isolate from infected teeth of a patient in University Hospital Tübingen, and propagated on S. epidermidis strain 1457 (49). S. aureus phages 11 and 80α (75) were used in the adsorption assay. Phage 187 (18) was used for the comparison of pBTn transduction efficiency with phage E72.

For the determination of growth properties of tested phages, a collection of S. epidermidis strains was used (Table 1). S. aureus strains 8325-4 (17) and RN4220 (51) and 35 field isolates of 7 CoNS, including *S. chromogenes*, S. hominis, *S. haemolyticus*, *S. petrasii*, S. sciuri, *S. simulans* and *S. xylosus*, were used in adsorption and/or host range assays. In addition to natural plasmids, the transposon plasmid pBTn, encoding chloramphenicol resistance (53), was used in a phage transduction assay. All the assays with pBTn were performed at 30°C to avoid loss of the plasmid. The pBTn was transferred to strain 1457 via electroporation, and this strain then served as the donor.

### Growth medium and phage propagation.

S. epidermidis phages were propagated on their propagation strains at 37°C on 1.5% meat peptone agar (MPA), prepared from 13 g nutrient broth CM0001 (Oxoid), 3 g yeast extract LP0021 (Oxoid), 5 g peptone LP0037 (Oxoid), 15 g agar LP0013 (Oxoid), and distilled water to a final volume of 1 liter (pH 7.4), overlaid with a top soft layer of MPA (0.7% agar) containing 2 mM CaCl_2_. The lysates were centrifuged at 5,000 × *g* for 15 min and filtered using a polyethersulfone membrane filter with a pore size of 0.45 μm (TPP, Switzerland).

### Morphological and proteomic characterization.

For electron microscopy, particles of phages E72, 27, 456, and 459 were purified in a CsCl density gradient (16). The transducing lysate of phage 48 was purified by fast protein liquid chromatography (FPLC) and ultrafiltration as described previously (76) with minor modifications. For the FPLC purification, a CIMmultus QA 8-ml monolithic column (Bia Separations, Slovenia) and NGC chromatography system (Bio-Rad, USA) were used. Phage lysate was mixed with 40 mM Tris buffer (pH 7.5) in a 1:1 ratio at a final NaCl concentration of 0.35 M. For ultrafiltration, Pellicon XL50 cassettes with a Biomax 300-kDa membrane (Millipore, USA) were used. Retentate and fractions containing phages were desalted and concentrated using Amicon Ultra 0.5-ml centrifugal filters (Millipore, USA) with a 100-kDa cutoff. Phages were diluted to an *A*_280_ of 0.5 to 1.0, and negatively stained samples were prepared by applying 4 μl of the diluted sample onto copper grids coated with a 12-nm carbon layer, stained with 2% uranyl acetate. Samples were observed using a Tecnai F20 electron microscope (Thermo Fisher Scientific, USA) operated at 200 kV with ×50,000 magnification.

Structural proteins of phage 48 and phage 48-derived small-headed particles purified in a CsCl gradient were determined by LC-MS/MS analysis performed using the Ultimate 3000 RSLCnano system (Thermo Fisher Scientific, USA) connected to an Impact II Qq time-of-flight mass spectrometer (Bruker, Bremen, Germany) as described previously (64). An in-house database of proteins encoded by strain SE48 and phage 48 was used for the final search. Only proteins that were identified based on at least two peptides were reported. Detected bacterial metabolic proteins were filtered out from the final report.

### Horizontal gene transfer experiments.

For transduction experiments, high-titer phage lysates were prepared in meat-peptone broth (MPB) prepared from 13 g of nutrient broth (Oxoid), 3 g yeast extract (Oxoid), 5 g peptone (Oxoid), and distilled water to a final volume of 1 l (pH 7.4). Alternatively, tryptone soy broth (TSB) (CM0129; Oxoid) was used. Transduction experiments were carried out as described previously (29). Transductants were selected on tryptone soy agar (TSA; CM0131; Oxoid) plates supplemented with sodium citrate (20 mM) and streptomycin (20 mg/liter), tetracycline (5 mg/liter), ampicillin (100 mg/liter), or chloramphenicol (10 mg/liter). The transduction of SeCI_SE48_ was performed without antibiotic selection. Plates were incubated at 37°C (30°C for strains with the plasmid pBTn) for 48 to 72 h. The transductants were verified by colony PCR assays using OneTaq Quick-Load 2× master mix with standard buffer (New England Biolabs, Ipswich, MA, USA) and a 200 nM concentration of each primer (Table 4) (77, 78). The reaction conditions were as follows: 94°C for 30s, followed by 30 cycles of denaturation at 94°C for 40 s, annealing at 53°C for 30 s, and extension at 68°C for 60 s per kb.

**TABLE 4:** PCR assays for verification of plasmid and SeCI_SE48_ transductions, SeCI_SE48_ packaging by S. epidermidis phage 48, and identification of prophage integration sites

Construct(s) detected	Target gene(s)	Source sequence	Forward primer	Reverse primer	Product length (bp)	Reference
Tetracycline resistance gene	*tetK*	pSE27_T	TCGATAGGAACAGCAGTA	CAGCAGATCCTACTCCTT	169	77
Chloramphenicol resistance gene	*cat*	pBTn	GCGACGGAGAGTTAGGTT	GCCTATCTGACAATTCCTGA	413	78
Streptomycin resistance gene	*aad(6)*	pSE27_S	ACGTTGAGACACTCCAAAACTC	AAATTATTGCTCTCGAGGGTTCA	429	This study
Plasmid pSE48_2	*rep*	pSE48_2	TTGAGCAAGAGGACGACCAA	AAATGCTACCCTTCGGCTCG	659	This study
S. epidermidis chromosome	*groEL*	S. epidermidis 1457	TCAGCGTTACAACATGCAGC	GTTGTCTTTCATAGTTGTATGTGCC	555	This study
S. epidermidis chromosome	*mer*	S. epidermidis SE48	ATTCGCACGTGAACCAGTGT	ACATGCTGCTGGTCACGG	343	This study
Integrated PICI	*groEL*, *int*	S. epidermidis SE48	TCAGCGTTACAACATGCAGC	GCACTCATTCCGTCACACAC	390	This study
Integrated PICI	*groEL*, *int*	S. epidermidis SE48	TCAGCGTTACAACATGCAGC	CGTGCAGGCGAGTTGTTAG	734	This study
SeCI_SE48_	*gp12*, *gp13*	SeCI_SE48_	CGTTGAGGGCTTGAAATGGG	GCACCTAACACTTGGCGTTT	758	This study
Packaged SeCI_SE48_	*gp22*, *int*	SeCI_SE48_	GTCCATACAAGTTAAACGGCGA	GCACTCATTCCGTCACACAC	665	This study
Phage 27 and 48 genomes	*dut*	Phage 48	AGGTGTATCGCAAAGCAGAGTT	TCTAACGGCTTACCTGGTTTCT	266	This study
Phage 456, 459, and E72 genomes	*dnaB*	Phage 459	GGACCAAGCACAAATGACACC	TGCTCAATCCCTCCTGCTTC	282	This study
Prophages 27, 48, 456, 459, and E72	*mer*, *int*	S. epidermidis NCTC 13924	ATTCGCACGTGAACCAGTGT	ACAGACGAAACAATCGCAGA	530	This study

Plasmid DNA was isolated from the transductants with a NucleoSpin plasmid kit (Macherey-Nagel, Düren, Germany) according to the manufacturer’s protocol using prolonged lysis with lysostaphin as described before (78). Plasmid DNA was analyzed by agarose gel electrophoresis in 1.2% agarose gel (Serva) in 1× Tris-acetate-EDTA (TAE) buffer. SmaI (New England BioLabs, Ipswich, MA, USA) macrorestriction analysis by pulsed-field gel electrophoresis (PFGE) was performed using a CHEF Mapper XA system (Bio-Rad) to confirm the transfer and integration of SeCI_SE48_.

### Electroporation.

Electroporation was conducted according to the transformation method for S. epidermidis described by Monk et al. (79). Briefly, overnight S. epidermidis cultures with an optical density at 600 nm (OD_600_) of 0.5 were chilled on ice, washed in 10% glycerol, and 50 μl was aliquoted to each tube. Before electroporation, competent cells were resuspended in 50 μl of 10% glycerol and 500 mM sucrose. A volume of 5 μl plasmid pBTn DNA (500 ng/μl) was added to the bacterial cells, transferred to a 1-mm electroporation cuvette (Bio-Rad), and then pulsed at 21 kV/cm, 100 Ω, and 25 μF. TSB supplemented with 500 mM sucrose was added to the electroporated bacteria, incubated at 30°C for 1 h before plating on TSA plates with chloramphenicol (10 mg/liter), and incubated at 30°C for 48 h.

### Lysogenization.

S. epidermidis strains SE27, SE48, SE456, SE459, and 1457 were lysogenized with phages 27, 48, 456, 459, and E72, respectively, as described previously (23). Integration sites were identified by PCR (Table 4) and the obtained amplicons were sequenced by Eurofins Genomics (Germany) using the same primers as in the PCR assay.

### Determination of host range, phage adsorption, and one-step growth curve.

Phage lysates were spotted onto the examined strains at 10×, 100×, and 1,000× routine test dilutions (RTD) (80). Productive phage infection was confirmed by the formation of single plaques. Adsorption curves of the phages were determined as described previously (81) using an input ratio (IR) of 10. The burst size of phage 48 on bacterial strains 1457 and 1457(SeCI_SE48_) was determined from the one-step growth curve as described previously (82) with minor modifications. Briefly, overnight bacterial culture was cultivated to an OD_600_ of 0.5 (10^8^ CFU/ml). Bacterial cultures were synchronized at 10°C for 30 min. The phage lysate was mixed with the bacterial culture at an IR of 0.01, and the mixture was incubated at 37°C. The titer of free phage particles was determined after 0 to 90 min at 5-min intervals.

### Statistical analysis.

Plasmid transduction efficiency and phage 48 burst size comparisons were evaluated by statistical analysis using the *t* test. A *P* value of 0.05 was used as the threshold for statistical significance.

### Genome analysis.

For whole-genome sequencing on the Illumina platform, the phage DNA was isolated using a phenol-chloroform method (83), and bacterial DNA was isolated from cultures cultivated in MPB using a High Pure PCR template preparation kit (Roche Diagnostics, Mannheim, Germany) according to the manufacturer’s instructions with the modification of adding lysostaphin (40 μg/ml, Sigma-Aldrich) for cell lysis. The 500-bp sequencing library was prepared with a NEBNext Ultra II DNA library prep kit for Illumina (New England BioLabs). The samples were sequenced using a MID output cartridge in a 150-bp paired-end mode on an Illumina NextSeq sequencing platform (Illumina, San Diego, CA, USA). The quality of sequencing reads was analyzed with FastQC v0.11.8 (84). Bases of lower quality and adapters were trimmed using the sliding window model in Trimmomatic Galaxy v0.36.5 (85).

For sequencing on an Oxford Nanopore platform, the bacterial DNA was isolated using the following steps. First, 10 ml of stationary overnight bacterial culture cultivated in TSB at 37°C was centrifuged at 3,000 × *g* and 10°C for 10 min, washed with 5 ml of wash solution (10 mM Tris-HCl, 10 mM EDTA, 10 mM EGTA, 1 M NaCl [pH 7.5]), and resuspended in Tris-EDTA (TE) buffer with lysostaphin (50 μg/ml), achromopeptidase (1,000 U/ml; Sigma-Aldrich), mutanolysin (40 U/ml; Sigma-Aldrich), and RNase A (200 μg/ml; New England BioLabs) in a total volume of 500 μl, followed by incubation for 1 to 2 h at 37°C until lysis appeared. Then, 30 μl of 10% SDS and 5 μl of proteinase K (20 mg/ml; Sigma-Aldrich) were added, and the sample was incubated for 60 min at 50°C, followed by heat inactivation for 10 min at 95°C and centrifugation at 9,700 × *g* for 5 min. DNA was isolated from the supernatant using the phenol-chloroform method (83). The library was prepared using an SQK-RBK004 rapid barcoding kit (Oxford Nanopore Technologies, UK) according to the manufacturer’s instructions. Libraries were sequenced with FLO-MIN106 flow cells (R9.4.1) on a MinION device (Oxford Nanopore Technologies, UK). The device was controlled with the software MinKNOW v.2.2.12 (Oxford Nanopore Technologies, UK). Base calling and demultiplexing were performed using Guppy v2.3.5. Reads were trimmed using fastp Galaxy v0.20.1 (86).

Complete genomes were obtained using Unicycler Galaxy v0.4.8.0 (87), including a hybrid assembly of bacterial genome sequences. Genomes were annotated using RAST (88) with manual inspection by BLAST (http://blast.ncbi.nlm.nih.gov/), InterPro (89), Phobius (90), and HHpred (91), which was also used for the secondary structure alignment of putative capsid assembly proteins. The Comprehensive Antibiotic Resistance Database (CARD), the NCBI database, ResFinder, and the virulence factor database (VFDB), accessed through ABRicate Galaxy v1.0.1 (92), were used to search for antibiotic resistance and virulence factor genes with ≥80% nucleotide identity and ≥60% length coverage. The genomic alignments were constructed using Easyfig v2.4 (93) and GView (94). Phage genome relatedness was calculated by OAT v 0.93.1 (95). The phylogenetic trees were built with the online tool Ngphylogeny.fr (96) as follows. The alignments were computed with Clustal Omega, and low-quality alignments were removed with BMGE (Block Mapping and Gathering with Entropy) software; the trees are the results of the maximum-likelihood-based inference of phylogenetic trees with Smart Model Selection software, and the statistical test for branch support was SH-like aLRT. The EMBOSS tools Needle (https://www.ebi.ac.uk/Tools/psa/emboss_needle/) and Stretcher (https://www.ebi.ac.uk/Tools/psa/emboss_stretcher/) were used for global pairwise sequence comparisons. The heat map was generated using R v3.6.3 (R Core Team). Sequencing reads mapping statistics were computed in CLC Genomic Workbench v3.6.5 (Qiagen Bioinformatics, Denmark). Physical termini of phage and SeCI_SE48_ genophores packaged in the virion capsid were determined from the alignment of Illumina reads with the PhageTerm tool (97). Multilocus sequence typing was performed using MLST v2.0.4 with database version 2.0.0 (98). The assignment of multilocus sequence types into clonal complexes was performed with eBURST v3 (99).

For comparative genomic analyses, the following genomes were used: phages PH15 and CNPH82 (36), CNPx (61), IPLA5 and IPLA7 (38), 71 and X2 (43), ETA (44), NM4 (45), and 80α (46); chromosomal islands of *S. lugdunensis* strain VISLISI_27 (100) and *S. haemolyticus* strain SGAir0252 (101); S. aureus pathogenicity islands SaPI1 (25), SaPI2R (102), SaPI3 (103), SaPI_N315_ (104), SaPI_IVM10_, SaPI_J11_, and SaPI_No.1_ (105), SaPI68111 (106), and SaPIeq1 (107); S. aureus resistance island SaRIfusB (108); S. epidermidis resistance islands SeRIfusB-704, SeRIfusB-2793, SeRIfusB-5907, SeRIfusB-7778 (14), and SeRIfusB-3692 (12); and S. epidermidis pathogenicity islands SePIfusB-857 (12) and SePI of strain 14.1.R1 (109).

### Data availability.

The sequences have been deposited in the GenBank database under the following accession numbers: phage 27 as vB_SepS_27, MW364971; phage 48 as vB_SepS_48, MW364972; phage 456 as vB_SepS_456, MW364973; phage 459 as vB_SepS_459, MW364974; phage E72 as vB_SepS_E72, MW364975; packaged phage-inducible chromosomal island SeCI_SE48_, MW368309; S. epidermidis strain SE48, NZ_CP066303; its plasmids pSE48_1, NZ_CP066304, and pSE48_2, NZ_CP066305; pSE27_S, MW364976; pSE27_T, MW364977; pSE456_1, MW364978; pSE456_2, MW364979; pSE456_3, MW364980; pSE456_4, MW364981; pSE459_1, MW364982; pSE459_2, MW364983; pSE459_3, MW364984; and pSE459_T, MW364985.
