# Supplementary material for: Staphylococcus epidermidis Phages Transduce Antimicrobial Resistance Plasmids and Mobilize Chromosomal Islands
Source: mSphere. 2021 May 12;6(3):e00223-21. doi: 10.1128/mSphere.00223-21 (PMC8125051; doi:10.1128/mSphere.00223-21)
Supplement: TABLE S1 [file mSphere.00223-21-st001.pdf]

Table S1: Mass spectrometry identification of proteins forming particles of phage 48 propagated on *S. epidermidis* strain SE48.

Protein	Source	Gene	MW (kDa)	calc. pI	SAF	NSAF (%)	Sum (Coverage)	Sum (# Peptides)	Sum (# Unique Peptides)
major capsid protein	phage 48	*gp7*	30	5,4	0,67	43,48	67%	18	18
capsid scaffolding protein	phage 48	*gp6*	22	4,7	0,38	24,83	57%	12	12
portal protein	phage 48	*gp3*	56	4,8	0,14	9,14	61%	25	25
capsid morphogenesis protein	$SeCI_{SE48}$	*gp14*	28	5,1	0,12	7,74	66%	12	12
receptor-binding protein	phage 48	*gp19*	97	7,1	0,02	1,40	23%	14	14
lower tail fiber	phage 48	*gp20*	58	5,1	0,03	1,92	24%	10	10
YopX-like protein	phage 48	*gp58*	16	4,5	0,07	4,35	39%	4	4
minor capsid protein	phage 48	*gp4*	37	8,6	0,02	1,44	18%	5	5
tail-associated lysin	phage 48	*gp18*	70	8,7	0,01	0,53	10%	5	5
Gam-like nuclease inhibitor	phage 48	*gp40*	18	5,5	0,02	1,62	42%	4	4
adaptor protein	phage 48	*gp9*	12	6,0	0,03	1,88	44%	3	3
ssDNA binding protein	phage 48	*gp42*	21	6,8	0,02	1,05	23%	3	3
ERF-like single-strand annealing protein	phage 48	*gp41*	24	5,5	0,01	0,63	14%	2	2

Structural proteins of phage and phage-derived small-headed particles purified in CsCl gradient were determined by LC-MS/MS analysis. (N)SAF - (normalized) spectral abundance factor; Sum (Coverage) - percentage of protein sequence covered by identified peptides; Sum (#Peptides) - number of peptides assigned to protein group, including peptides that are shared with other protein groups; Sum (#Unique peptides) - number of peptides that are unique to the specific protein group.
