# Supplementary material for: Staphylococcus epidermidis Phages Transduce Antimicrobial Resistance Plasmids and Mobilize Chromosomal Islands
Source: mSphere. 2021 May 12;6(3):e00223-21. doi: 10.1128/mSphere.00223-21 (PMC8125051; doi:10.1128/mSphere.00223-21)
Supplement: TABLE S2 [file mSphere.00223-21-st002.pdf]

Table S2: Annotation of predicted *S. epidermidis* phage 48-encoded proteins compared to phages 27, 456, 459 and E72.

Phage 48				Phage 27		Phage 456		Phage 459		Phage E72	
**module**	**gene**	**putative product**	**length (aa)**	**gene**	**identity %**	**gene**	**identity %**	**gene**	**identity %**	**gene**	**identity %**
structural proteins	*gp1*	terminase small subunit (TerS)	126	*gp1*	100.0	*gp1*	22.2	*gp1*	22.2	*gp1*	100.0
	gp2	terminase large subunit (TerL)	421	*gp2*	99.5	*gp2*	98.8	*gp2*	99.5	*gp2*	97.6
	gp3	portal protein (Prt)	478	*gp3*	99.0	*gp3*	99.0	*gp3*	99.0	*gp3*	98.5
	gp4	minor capsid protein	317	*gp4*	96.5	*gp4*	96.5	*gp4*	96.5	*gp4*	96.5
	gp5	hypothetical protein	68	*gp5*	82.4	*gp5*	82.4	*gp5*	82.4	*gp6*	82.4
	gp6	capsid scaffolding protein (Csp)	197	*gp6*	30.1	*gp6*	30.1	*gp6*	30.1	*gp7*	47.1
	gp7	major capsid protein (Mcp)	276		ns		ns		ns	*gp8*	99.6
	gp8	Rho termination factor	96	*gp8*	96.9	*gp8*	96.9	*gp8*	96.9	*gp9*	99.0
	gp9	adaptor protein	104	*gp9*	98.1	*gp9*	98.1	*gp9*	98.1	*gp10*	98.1
	gp10	stopper protein	109	*gp10*	99.1	*gp10*	99.1	*gp10*	99.1	*gp11*	98.2
	gp11	tail complementation protein	137	*gp11*	98.5	*gp11*	98.5	*gp11*	98.5	*gp12*	98.5
	gp12	tail terminator	145	*gp12*	93.1	*gp12*	93.1	*gp12*	93.1	*gp13*	93.8
	gp13	major tail protein (Mtp)	179	*gp13*	99.4	*gp13*	99.4	*gp13*	99.4	*gp14*	98.3
	gp14	tail assembly chaperone	164	*gp14*	100.0	*gp14*	100.0	*gp14*	100.0	*gp15*	98.8
	gp15	tail assembly chaperone	100	*gp15*	89.0	*gp15*	89.0	*gp15*	89.0	*gp16*	89.0
	gp16	tape measure protein (Tmp)	1034	*gp16*	96.8	*gp16*	94.3	*gp16*	96.8	*gp17*	93.8
	gp17	distal tail protein (Dit)	312	*gp17*	96.5	*gp17*	99.0	*gp17*	96.5	*gp18*	97.8
	gp18	tail-associated lysin (Tal)	618	*gp18*	99.4	*gp18*	98.5	*gp18*	99.4	*gp19*	99.0
	gp19	receptor-binding protein (Rbp)	888	*gp19*	97.8	*gp19*	97.2	*gp19*	97.8	*gp20*	97.3
	gp20	lower tail fiber protein (FibL)	510	*gp20*	98.8	*gp20*	98.8	*gp20*	98.8	*gp21*	98.6
	gp21	tail fiber assembly protein	112	*gp21*	98.2	*gp21*	98.2	*gp21*	98.2	*gp22*	98.2
	gp22	tail chaperone	46	*gp22*	100.0	*gp22*	100.0	*gp22*	100.0	*gp23*	100.0
	gp23	holin regulator	99	*gp23*	100.0	*gp23*	100.0	*gp23*	100.0	*gp24*	78.8
	gp24	cell wall hydrolase (Hyd)	633	*gp24*	98.0	*gp24*	97.6	*gp24*	94.2	*gp25*	95.3
	gp25	upper tail fiber protein (FibU)	169	*gp25*	97.0		ns	*gp25*	97.0	*gp26*	98.8
	gp26	hypothetical protein	175	*gp26*	99.4		ns	*gp26*	99.4	*gp27*	98.9
lytic proteins	*gp27*	holin (Hol)	89	*gp27*	86.5	*gp28*	86.5	*gp27*	84.6	*gp28*	87.6
	gp28	amidase (Ami)	467	*gp28*	50.7	*gp29*	50.7	*gp28*	50.7	*gp29*	51.2
lysogenic conversion	*gp29*	hypothetical protein	37		ns		ns		ns		ns
	gp30	DNA binding protein	73		ns		ns		ns		ns
	gp31	hypothetical protein	65		ns		ns		ns		ns
lysogeny regulation	*gp32*	integrase (Int)	458	*gp30*	96.5	*gp31*	96.3	*gp30*	96.3	*gp33*	96.5
	gp33	DUF5067 domain protein	271	*gp31*	100.0	*gp32*	21.4	*gp31*	100.0	*gp34*	20.8
	gp34	ImmA/IrrE endopeptidase	156	*gp32*	100.0	*gp33*	46.2	*gp32*	100.0	*gp36*	46.8
	gp35	CI-like repressor (CI)	104	*gp33*	100.0		ns	*gp33*	100.0	*gp37*	28.4
	gp36	Cro-like repressor (Cro)	85	*gp34*	100.0		ns	*gp34*	98.8		ns
	gp37	antirepressor (Ant)	246	*gp35*	100.0		ns	*gp35*	99.6		ns
DNA metabolism	*gp38*	hypothetical protein	31	*gp36*	100.0	*gp41*	78.4	*gp36*	100.0	*gp42*	83.8
	gp39	hypothetical protein	83	*gp37*	100.0		ns		ns		ns
	gp40	Gam-like nuclease inhibitor (Gam)	161	*gp38*	100.0		ns		ns		ns
	gp41	ERF-like single strand annealing protein (Erf)	207	*gp39*	100.0		ns		ns		ns
	gp42	single stranded DNA binding protein (Ssb)	186	*gp40*	100.0	*gp45*	30.1	*gp40*	31.2	*gp46*	31.7

Table S2: Annotation of predicted *S. epidermidis* phage 48-encoded proteins compared to phages 27, 456, 459 and E72. (continued)

Phage 48				Phage 27		Phage 456		Phage 459		Phage E72	
**module**	**gene**	**putative product**	**length (aa)**	**gene**	**identity %**	**gene**	**identity %**	**gene**	**identity %**	**gene**	**identity %**
DNA metabolism	*gp43*	HNHc nuclease (Hnh)	229	*gp41*	100.0	*gp46*	72.9	*gp41*	75.1	*gp47*	83.8
	gp44	XRE family transcriptional regulator	76	*gp42*	100.0		ns	*gp42*	98.7		ns
	gp45	transcription factor	257	*gp43*	100.0		ns		ns		ns
	gp46	DNA replication protein DnaD	259	*gp44*	100.0		ns		ns		ns
	gp47	replicative DNA helicase DnaB	256	*gp45*	99.6		ns		ns		ns
	gp48	host DNA synthesis inhibitor Sri	53	*gp46*	100.0		ns		ns		ns
	gp49	DUF3269 domain protein	81	*gp47*	100.0	*gp51*	98.8		ns	*gp52*	96.3
	gp50	Holliday junction resolvase (RusA)	135	*gp48*	100.0		ns		ns	*gp53*	100.0
	gp51	hypothetical protein	63	*gp49*	100.0		ns	*gp51*	88.9	*gp54*	93.7
	gp52	PVL ORF-50-like protein	119	*gp50*	100.0	*gp54*	95.0	*gp52*	95.0	*gp55*	98.3
	gp53	nucleotide kinase	150	*gp51*	100.0	*gp55*	69.9	*gp53*	39.8	*gp56*	70.6
	gp54	hypothetical protein	95	*gp52*	100.0		ns		ns		ns
	gp55	HNH homing endonuclease	226	*gp53*	100.0	*gp56*	95.6	*gp54*	97.4	*gp57*	93.8
	gp56	hypothetical protein	64	*gp54*	100.0	*gp57*	85.9	*gp55*	77.3	*gp58*	87.7
	gp57	hypothetical protein	87	*gp55*	100.0		ns		ns		ns
	gp58	YopX-like protein	135	*gp56*	100.0		ns		ns		ns
	gp59	hypothetical protein	55	*gp57*	100.0	*gp59*	23.8	*gp56*	23.2	*gp62*	54.4
	gp60	hypothetical protein	59	*gp58*	100.0	*gp60*	93.2	*gp58*	94.9	*gp63*	33.3
	gp61	dUTP diphosphatase (Dut)	175	*gp59*	100.0		ns		ns		ns
	gp62	membrane-associated protein	77	*gp60*	100.0	*gp62*	96.1	*gp60*	98.7		ns
	gp63	hypothetical protein	83	*gp61*	100.0	*gp63*	96.4	*gp61*	100.0		ns
	gp64	transcription activator RinB	56	*gp62*	100.0	*gp64*	91.1	*gp62*	89.3	*gp67*	85.7
	gp65	membrane-associated protein	72	*gp63*	100.0		ns		ns		ns
	gp66	membrane-associated protein	46	*gp64*	97.8	*gp67*	87.0	*gp64*	87.0	*gp69*	87.0
	gp67	hypothetical protein	72	*gp65*	100.0		ns		ns	*gp70*	97.2
	gp68	transcriptional regulator RinA	138	*gp66*	100.0	*gp68*	99.3	*gp65*	99.3	*gp71*	100.0

ns - no significant similarity (E value > 0.0001); the abbreviations used in this work are given in parentheses after the product name. Identity value is based on Emboss Needle optimal global sequence alignment.
