# Supplementary material for: Staphylococcus epidermidis Phages Transduce Antimicrobial Resistance Plasmids and Mobilize Chromosomal Islands
Source: mSphere. 2021 May 12;6(3):e00223-21. doi: 10.1128/mSphere.00223-21 (PMC8125051; doi:10.1128/mSphere.00223-21)
Supplement: TABLE S3 [file mSphere.00223-21-st003.pdf]

Table S3: Transfer of plasmid pBTn via electroporation and transduction mediated by phages 187 and E72.

S. epidermidis strain	ST type	Transfer via electroporation	Transduction rate	
			phage 187	phage E72
E12	19	ns	ns	ns
E14	new type	ns	ns	ns
E16	457	ns	ns	ns
E18	65	ns	1.0×10^{-8}	4.3×10^{-6}
E20	153	ns	7.0×10^{-8}	5.1×10^{-8}
E21	130	yes	1.0×10^{-6}	6.6×10^{-8}
E23	795	ns	ns	1.3×10^{-4}
E24	73	ns	1.3×10^{-7}	ns
E28	5	ns	ns	ns
E30	5	yes	ns	ns
E31	5	ns	ns	1.6×10^{-6}
E33	640	ns	ns	1.7×10^{-6}
E34	new type	yes	ns	1.9×10^{-6}
E43	327	yes	5.0×10^{-9}	1.3×10^{-4}
E44	new type	yes	5.2×10^{-8}	ns
E46	944	yes	ns	1.3×10^{-4}
E47	89	ns	6.0×10^{-8}	ns
E53	2	yes	ns	6.7×10^{-8}
E55	new type	ns	ns	ns
E57	5	ns	1.8×10^{-8}	ns
E63	66	ns	ns	1.3×10^{-4}
E64	5	yes	1.0×10^{-8}	1.8×10^{-6}
E65	5	ns	ns	ns
E66	5	yes	3.0×10^{-7}	ns
E72	5	ns	ns	1.1×10^{-8}
E77	73	ns	5.0×10^{-9}	3.7×10^{-8}
E88	2	ns	ns	4.0×10^{-6}
E89	5	yes	ns	ns
E90	new type	ns	ns	3.3×10^{-6}
E91	5	ns	ns	1.1×10^{-7}
E95	35	yes	ns	8.4×10^{-8}
E97	736	ns	1.8×10^{-8}	2.6×10^{-6}
E98	5	ns	ns	2.3×10^{-7}
E102	130	ns	ns	1.8×10^{-5}
E103	130	ns	ns	ns
E108	new type	ns	ns	ns
E113	981	ns	ns	ns

The ratio of transduction is presented as the number of transductants per ml phage lysate relative to the transducing phage titer; each transduction was carried out in triplicates and the average rate is provided. ns – transfer not successful.
